# Coerción en las hospitalizaciones psiquiátricas en Chile: El sufrimiento de la locura en el siglo XXI

**DOI:** 10.18294/sc.2023.4349

**Published:** 2023-07-27

**Authors:** Manuel Alejandro Castro

**Affiliations:** 1 Doctor en Sociología. Académico, Departamento de Trabajo Social, Universidad Alberto Hurtado, Santiago, Chile. alejandrocastroharrison@gmail.com Universidad Alberto Hurtado Departamento de Trabajo Social Universidad Alberto Hurtado Santiago Chile alejandrocastroharrison@gmail.com

**Keywords:** Salud Mental, Hospitalización, Coerción, Chile, Mental Health, Hospitalization, Coercion, Chile

## Abstract

Este artículo aborda el problema de la coerción en las hospitalizaciones psiquiátricas chilenas desde la perspectiva de personas usuarias que participan en un tratamiento en salud mental en la red pública de atención. Entre 2019 y 2020 se realizó un estudio cualitativo con enfoque epistémico hermenéutico, en el que se entrevistaron 25 personas de ambos sexos (15 hombres y 10 mujeres) con diagnóstico psiquiátrico, con el fin de analizar sus relatos y repensar críticamente las prácticas de intervención que se desarrollan al interior de las hospitalizaciones psiquiátricas en Chile, las cuales se constituyen como un espacio de importancia para la salud pública chilena y los derechos de las personas con problemas de salud mental. Uno de los principales hallazgos es que, en desmedro de la recuperación de las personas usuarias, las prácticas coercitivas siguen manteniéndose en Chile, lo que implica un impacto negativo en la calidad de vida y en la libertad ciudadana de las personas con problemas de salud mental.

## Abstract (en)

This article reflects on the problem of coercion in Chilean psychiatric hospitalizations from the perspective of users who participate in mental health treatment programs in the public healthcare system. Qualitative research from a hermeneutical epistemic approach was carried out between 2019 and 2020, which included 25 interviews with individuals of both sexes (15 men and 10 women) who had a psychiatric diagnosis. The purpose of this research was to analyze their narratives and critically reframe intervention practices implemented in psychiatric hospitalizations in Chile, which constitute an important space for public health in the country as well as the rights of people with mental health problems. One of the principal findings of the study was that coercive practices still persist in Chile, despite being detrimental to the recovery of mental health care users, representing a negative impact on the quality of life and citizen freedoms of individuals with mental health problems.

## INTRODUCCIÓN

El lugar de las hospitalizaciones psiquiátricas siempre ha sido un espacio polémico al interior de la psiquiatría y la salud mental^1^. Estudios recientes^2^^,^^3^^,^^4^^,^^5^^,^^6^ han mostrado esta tensión a partir de las experiencias de los pacientes psiquiátricos en estos espacios terapéuticos. Gran parte de estas investigaciones han dado cuenta de que existen problemas con la incorporación del enfoque definido por la Ley 21.331^7^, de derecho y equidad, trato digno y escucha activa entre los equipos de salud mental y usuarios en las hospitalizaciones. Esto ya se ha venido problematizando desde la década de 1960 con los estudios de Goffman^8^, y ha sido retomado como una problemática a nivel mundial por investigadores como Fennel^9^ y Mills^10^, desde la perspectiva de la coerción.

En Chile, la red de salud mental cuenta con una serie de unidades de hospitalización psiquiátrica que no han estado ajenas a este problema, por lo que el Estado chileno promulgó la Ley 21.331^7^ que, en el Título III, define a la hospitalización psiquiátrica “como una medida terapéutica excepcional y esencialmente transitoria”^7^. Sin embargo, allí se reconoce que es una práctica que afecta el derecho a la libertad de las personas (Artículo 13), por lo que intenta regular esta intervención a partir del enfoque de derechos, “garantizando los derechos humanos de las personas con enfermedad mental o discapacidad psíquica”^7^.

Las unidades de hospitalización de cuidados intensivos psiquiátricos (UHCIP) es la denominación que se le da en la actualidad a las unidades de hospitalización psiquiátrica en Chile^11^. Estos dispositivos, pertenecientes a la red de salud mental comunitaria^12^, son los lugares encargados de realizar la estabilización psicopatológica de las personas con enfermedad psiquiátrica “que en algún momento de su enfermedad presentan descompensación de su cuadro clínico, constituyendo riesgo para sí mismo o para terceros (incluso vital)”^11^ y que, por tanto, necesitan cuidados intensivos para resolver sus problemas de salud mental en etapa de crisis. 

Así, el modelo de gestión de las UHCIP^11^, en el punto 13.3 sobre el ambiente laboral, señala que las unidades de hospitalización psiquiátrica son lugares “de alta intensidad y conmoción, tanto para personas hospitalizadas, como para el equipo, donde la seguridad está en tensión”^11^. De este modo, las experiencias satisfactorias de las personas usuarias de estos servicios dependen siempre de un “buen clima laboral y de relaciones terapéuticas positivas”^11^, por tanto, cuando estas condiciones no se dan, tendrán consecuencias en las experiencias de esas personas usuarias y en su salud, que repercutirán en los equipos que conforman estas unidades. 

Esto último ha sido corroborado por distintas entidades tales como la Convención sobre el Derecho de las Personas con Discapacidad, en el informe para Chile del año 2016^13^, en el que indica la preocupación sobre las prácticas de coerción existentes en estos lugares. Algo corroborado por el Informe de Derechos Humanos de la Universidad Diego Portales del año 2018^14^, y que la Corte Suprema de Chile ratificó como sospecha en el oficio N.º 164-2018^15^, en relación con lo que ocurre al interior de estas unidades de cuidados psiquiátricos. 

Dado lo anterior, resulta importante reflexionar críticamente acerca de las prácticas de intervención que se desarrollan al interior de las hospitalizaciones psiquiátricas en Chile, ya que se constituyeron en un espacio de importancia para la salud pública chilena y los derechos de las personas con problemas de salud mental, los que han ido adquiriendo una importancia sustantiva y creciente en el último tiempo y es de prever que será de mayor importancia en el futuro, dado los movimientos sociales que han surgido en Chile y América Latina^16^.

Las denuncias realizadas a ciertos hospitales psiquiátricos o unidades de hospitalizaciones por distintos grupos de personas usuarias de la psiquiatría y el Instituto Nacional de Derechos Humanos^17^, han ido aumentando en el transcurso del tiempo, por los problemas de coerción que han ocurrido en estos dispositivos^14^, y que la Ley 21.331 intenta regular^7^.

Los esfuerzos que se han efectuado en el sistema público de atención en salud mental para incorporar el enfoque de derechos, en general, han sido estructurales y fundados en la teoría y en aspectos normativos^12^^,^^18^. No obstante, existen algunas investigaciones situadas^2^^,^^3^^,^^4^^,^^17^ que permiten fundar los procesos de construcción de una nueva cultura de derechos en las prácticas de intervención de las instituciones psiquiátricas y de salud mental en nuestro país.

En esa línea, la idea principal de este trabajo es reflexionar acerca de las prácticas de intervención y de coerción con las personas hospitalizadas, al interior de las UHCIP (incluyendo los hospitales psiquiátricos). Ello quiere decir que tanto los procedimientos, las intervenciones profesionales como las prácticas cotidianas al interior de estos dispositivos tienen dimensiones coercitivas que se expresan en los relatos de las personas usuarias que han experimentado la hospitalización psiquiátrica en Chile. De esa manera, a la luz de las voces de las personas usuarias, se intenta repensar un enfoque que asegure el trato digno, la escucha activa y la comunicación asertiva en esta fase del tratamiento psiquiátrico agudo, como preocupación fundamental del cuidado.

### Las hospitalizaciones psiquiátricas

Las hospitalizaciones psiquiátricas son estrategias de intervención terapéutica que se constituyen como parte del tratamiento en salud mental en un periodo determinado y acotado, con pacientes afectados por un problema psiquiátrico y que, por diferentes motivos, su gravedad es tratada en dispositivos de cuidados intensivos relacionados con el encierro^19^. Estas situaciones complejas o de gravedad, generalmente están asociadas a problemas psicopatológicos, el riesgo para sí mismos y/o para terceros, necesitando una unidad hospitalaria específica con competencias técnicas que permita la estabilización del cuadro psicopatológico y el cuidado del usuario.

En ese contexto, los estudios que se han realizado acerca de las unidades de hospitalización y las prácticas de intervención se centran generalmente en el problema de la coerción. En América Latina encontramos pocos trabajos al respecto. Uno de ellos es el estudio doctoral de Sara Ardiles^4^, quien revisa los relatos de mujeres en contextos de hospitalización psiquiátrica en Argentina y las narrativas de coerción en estos lugares. Por otro lado, en el estudio “Por el Derecho a la Locura: la reinvención de la Salud Mental en América Latina”^16^ se recopilan distintas experiencias de personas con problemas psiquiátricos en los dispositivos de salud mental. Se destacan las experiencias de Paraguay y Centroamérica, donde se denuncia el problema de la coerción y reclusión de las personas usuarias en las hospitalizaciones psiquiátricas. En el caso chileno, los estudios de Castro^2^^,^^3^ se centran en el sufrimiento psíquico y los efectos performativos de la coerción psiquiátrica en las hospitalizaciones de nuestro país.

En Europa, autores como Peter Gøtzsche^20^, Rafael Huertas^21^, Alberto Fernández Liria^22^ y Ortiz Lobo^23^ colocan nuevamente en escena el problema de la coerción psiquiátrica, cuestionando las prácticas de intervención en las unidades de hospitalización, a la luz de los derechos humanos de personas con problemas psiquiátricos.

A partir de la revisión de experiencias de investigación, principalmente de Europa y Oceanía, que se vinculan con las hospitalizaciones psiquiátricas y la coerción, se destaca el enfoque de derechos como eje articulador y principio ético fundamental, a propósito de los lineamientos del Comité sobre Derechos de Personas con Discapacidad (CRPD) de la Organización de las Naciones Unidas. Ejemplo de ello, es lo que ocurre en Noruega y Finlandia, donde se visibilizan las consecuencias de la reclusión y restricción de pacientes en las hospitalizaciones psiquiátricas^24^. Cabe señalar que en estos países las prácticas de coerción están prohibidas por razones éticas y, en esa línea, el estudio de Steintert^24^ da cuenta de la relación entre los pacientes sometidos a coerción, las prácticas clínicas y la escasa mejoría. En la misma línea, es posible identificar que, en Finlandia, se realizó una investigación sobre el impacto de la reclusión y coerción en las prácticas de intervención de las unidades de hospitalización de todo ese país^25^^,^^26^. En Dinamarca, se realizó otro trabajo sobre coerción psiquiátrica, relacionado con los procesos de intervención en las hospitalizaciones, en el que Andersen y Nielsen^27^ indican que el 28% de las personas ingresadas a las unidades de hospitalización fueron objeto de coerción con procedimientos forzosos durante las primeras horas. Como recomendación, los autores sugieren generar políticas integrales por parte del Estado para reducir estas prácticas en estos lugares. En el caso de Alemania, el estudio realizado por Flammer y Steinter^28^ describe las hospitalizaciones involuntarias y las prácticas coercitivas, objetando las contenciones mecánicas y farmacológicas y la gravedad de los incidentes violentos en las hospitalizaciones psiquiátricas. 

El 2017, en Inglaterra, se realizó un estudio cualitativo acerca de las experiencias de funcionarios y pacientes sobre la toma de decisiones en las hospitalizaciones psiquiátricas, y cómo afecta la relación terapéutica entre estos dos estamentos^29^. Este estudio muestra un trabajo de colaboración entre personas usuarias y trabajadoras de la unidad de hospitalización psiquiátrica, específicamente de Londres, con el fin de disminuir las prácticas coercitivas en las hospitalizaciones. En el mismo año, y también en Inglaterra, se realizó una investigación sobre la *Mental Health Act* del año 2005 y las implicancias en la salud mental inglesa, específicamente con respecto al problema de las prácticas de coerción psiquiátrica^30^. Este trabajo muestra que el 41% de las personas usuarias que habían cursado hospitalizaciones psiquiátricas relataron que recibieron medidas coercitivas por parte del personal de dichas unidades. 

Estos hallazgos se relacionan con un estudio australiano sobre las prácticas coercitivas de los trabajadores sociales en las unidades de hospitalización psiquiátrica^31^. En este mismo país, Fletcher^32^ parte de la base del reconocimiento de los problemas de coerción al interior de las hospitalizaciones, creando un proyecto de investigación aplicada a los *safewards,* y revela que las prácticas de intervenciones en ciertos pabellones de los servicios de hospitalización con un enfoque de derechos cambian sustantivamente la percepción de las personas usuarias acerca del trato digno. Finalmente, Sampogna^33^ indica en su investigación que las prácticas coercitivas se convierten en muy poco tiempo en abusos a los Derechos Humanos.

En Canadá, un estudio cualitativo, que realizó una revisión de las prácticas de coerción al interior de las unidades de hospitalización psiquiátrica con pacientes agudos, específicamente con personas con psicosis en primer episodio^34^, propone que explorar los sentimientos y emociones de las personas en periodos de hospitalización conduciría a una mejora en la relación terapéutica, reduciendo las prácticas de coerción. En la misma línea, en Holanda se realizó otro estudio sobre unidades de personas hospitalizadas en las que se aplica un programa de reducción de la reclusión y coerción psiquiátrica, y muestra que, al generar este tipo de prácticas de intervención, se reduce significativamente la coerción, generando un impacto positivo en las personas usuarias^35^. En Dinamarca, se realizó una investigación para identificar los factores de riesgo asociados a las medidas coercitivas, y así hallar las causas de la coerción en estas unidades^36^, y uno de los grandes hallazgos fue que las prácticas coercitivas aumentaban cuando las personas ingresaban por primera vez a este tipo de lugares, disminuyendo posteriormente en las siguientes hospitalizaciones. 

Volviendo a América Latina, Gooding y Mc Sherry^37^ insisten en que hay pocas investigaciones en este campo, y que los esfuerzos tanto en Perú como en Argentina han sido por la vía legal (algo que Chile abordó en el año 2021). Por ende, el esfuerzo de los Estados está más ligado a lo normativo y legal, que a la revisión de prácticas de coerción: “Los países latinoamericanos están dando claramente algunos pasos para avanzar en el enfoque de “voluntad, preferencia y derechos”^37^. En Chile, un estudio del año 2018 sobre internaciones involuntarias plantea el uso de la coerción en los procesos de internación y en las propias hospitalizaciones, evaluándolas como algo nocivo para las prácticas terapéuticas y basadas en derechos, que son el propósito fundamental de los cuidados que debiera tener la hospitalización psiquiátrica^38^. 

Gooding y Mc Sherry definen la hospitalización psiquiátrica como una reclusión y contención permitida “para controlar o gestionar los comportamientos de una persona”^37^ y, a propósito del informe de coerción psiquiátrica presentado a las Naciones Unidas en el año 2018, consideran que la coerción es un problema de derechos a nivel global. En consonancia con estos autores, el investigador y psiquiatra chileno Álvaro Barrera^39^ plantea que, al hospitalizar a una persona con un problema de salud mental se restringe la libertad, y por ende los derechos fundamentales, y como consecuencia se pueden vulnerar derechos humanos.

Una interesante investigación centrada en las prácticas de intervención de tipo coercitiva en España muestra las posturas tradicionales de psiquiatras que sostenían que las prácticas de intervención coercitivas eran parte del tratamiento^40^. La lógica del “*es por tu bien*”^40^ se constituye en una premisa fundamental de las prácticas de intervención en estos lugares. Las contenciones mecánicas y farmacológicas serían las principales medidas coercitivas que se toman con los usuarios, debiendo ser reguladas, a partir de las recomendaciones que hizo el Comité sobre los Derechos de las Personas con Discapacidad en 2016 a España, en materia de derechos humanos. 

Por otro lado, el estudio de Bootlender^41^ concuerda al indicar que la percepción de las personas que tienen hospitalizaciones, ya sea forzosa o no, y que sufren prácticas coercitivas, tienen una perspectiva negativa respecto al tratamiento psiquiátrico, básicamente por generar restricciones a la libertad de movimiento, el control físico y de administración de psicofármacos sin consentimiento. Esto último tendría correlación con otro estudio realizado en Chile sobre política criminal, relacionado con la aplicación de las terapias electroconvulsivas (TEC), las que son definidas como prácticas coercitivas, de tortura y otros tratos crueles en los hospitales psiquiátricos^42^. Este estudio indica que es el Instituto Nacional de Derechos Humanos el que debería hacerse cargo de este problema al interior de las unidades de hospitalización psiquiátrica. En esa misma línea, Rzhevskaya^43^ refiere que toda práctica de coerción es violencia psiquiátrica y muestra cómo los pacientes psiquiátricos son sometidos a coacción y violencia por parte del personal médico, haciendo hincapié en que el gran problema es la no inclusión de perspectivas de derechos de las personas usuarias en las prácticas de intervención de dichas unidades.

Por último, Clara Cocho^44^ aborda la cuestión de la satisfacción de las personas usuarias y los grandes obstáculos que las y los profesionales y técnicos de las unidades de hospitalización psiquiátrica colocan para poder aplicar cuestionarios y otros instrumentos que midan estos problemas, ya que no serían parte de sus funciones. Para Cocho, esto sería uno de los grandes impedimentos que tiene el enfoque de derecho para la instalación y relevamiento del trato digno de las personas con problemas de salud mental.

## ASPECTOS METODOLÓGICOS

Este trabajo se enmarca en la investigación doctoral sociológica “Los efectos performativos de la psiquiatría en la vida de las personas diagnosticadas psiquiátricamente: el sufrimiento de la locura”^2^ publicada el año 2021 en la Universidad Alberto Hurtado. Ella tiene un carácter descriptivo-comprensivo y de naturaleza cualitativa, basada en un enfoque epistémico hermenéutico y desarrollado a través de un análisis narrativo. Participaron voluntariamente 25 personas de ambos sexos diagnosticados psiquiátricamente (15 hombres y 10 mujeres), pertenecientes a la red pública de atención de salud mental chilena del Servicio de Salud Metropolitano Sur (SSMS) y Servicio de Salud Metropolitano Norte (SSMN). 

Las personas usuarias que colaboraron en esta investigación tenían asignados diagnósticos de esquizofrenia, trastorno afectivo bipolar y depresión severa, denominados como trastornos mayores. Contaban con cobertura pública a través de las Garantías Explicitas de Salud (GES), ley que permite el acceso, oportunidad, protección financiera y calidad en la atención en enfermedades catastróficas como la esquizofrenia, el trastorno afectivo bipolar y la depresión, consideradas así tanto en el sector público como privado. Esto último es de gran relevancia, ya que la gran mayoría de las personas que se adscriben a las GES en los SSMS y SSMN pertenecen a un estrato social bajo. Sin embargo, Chile no cuenta con estudios específicos sobre estratificación social y atención de salud mental, ya que es una política generalizada a toda la población, de acuerdo a la Ley GES.

En cuanto a los criterios de selección de las personas entrevistadas, debían ser mayores de 18 años y participar activamente en un tratamiento psiquiátrico de la red pública de salud mental de Chile; haber tenido la experiencia de haber sido hospitalizadas al menos una vez en los últimos 10 años en la red pública de salud mental chilena (UHCIP, tanto de hospitales generales como psiquiátricos); estar en tratamiento psicofarmacológico activo y con compensación psicopatológica; y contar con la confirmación diagnóstica de dos años como mínimo, dado que generaba una mayor amplitud en la posibilidad de acceder a la muestra. 

En lo referente con el análisis narrativo, todas las entrevistas fueron desarrolladas a través de índices temáticos y, en un segundo momento, codificadas a través del *software* ATLAS.TI. Este doble análisis permitió dar cuenta en dos momentos sobre los discursos narrativos^45^, permitiendo profundizar la experiencia del sujeto entrevistado.

El análisis de este trabajo se realizó principalmente desde una perspectiva narrativa temática con el objeto de recoger las experiencias de las personas que han sido diagnosticadas psiquiátricamente. Para ello, se realizaron entrevistas en profundidad sin reiteración, con el fin de poder comprender de una manera acabada la experiencia de estas personas en relación con sus vivencias. Una de las temáticas principales ha sido la experiencia en torno al sufrimiento psíquico que conlleva tener un diagnóstico relacionado con la salud mental, y su vínculo con la hospitalización psiquiátrica. De esa manera, la temática abordada por este trabajo, a diferencia de otros, se refiere principalmente a la experiencia que han tenido las personas diagnosticadas psiquiátricamente en relación con la hospitalización psiquiátrica y las prácticas de coerción que emergen en esta intervención.

De acuerdo a Susan Chase, la narrativa es “la creación de significado en retrospectiva, la configuración o el ordenamiento de la experiencia”^46^. De esa manera, se pueden alcanzar y comprender de una forma más acabada las experiencias personales de estos sujetos, comunicando el punto de vista del narrador, sus emociones, pensamientos e interpretaciones sobre su experiencia personal. Desde esta perspectiva, el narrador se vuelve un protagonista de una historia que está usurpada por el saber psiquiátrico y, así, la experiencia del “loco” se comprenderá desde su habilitación científica, es decir, el diagnóstico psiquiátrico. Entender la experiencia de vivir con un diagnóstico psiquiátrico es abrirnos a una narrativa que cuenta los acontecimientos perturbadores de la vida del “loco” pero, además, de cómo ello se ensambla en una vida social, más allá de los significados personales, ya que también revelaría una visión social y profundamente historizada de la locura. Ello tiene que ver con cómo los discursos psiquiátricos han transformado la vida de estas personas, moldeando hasta lo más oculto de la subjetividad humana que reside en esas experiencias. Lasllet dirá que estas narrativas personales nos pueden mostrar: “la acción y los significados individuales y colectivos, así como los procesos sociales por los cuales la vida social y las relaciones humanas son hechas y cambiadas”^45^. En definitiva, la experiencia pasa a ser una cuestión de mucha más profundidad hermenéutica que contar historias en sí, ya que también develan procesos más estructurales que se esconden en el interior del relato.

Este estudio se ajustó a los criterios elaborados por la Agencia Nacional de Investigación y Desarrollo (ANID) de Chile, y la aplicación de los consentimientos informados correspondientes de acuerdo a la resolución del comité de ética R-431 de la Universidad Alberto Hurtado y el Comité Asesor de Bioética de ANID del año 2019. Las personas entrevistadas participaron voluntariamente, con posterioridad a la explicitación de la confidencialidad y anonimato, además del objetivo de la investigación. Toda la información recolectada fue protegida, identificando a las personas entrevistadas con números correlativos.

## COERCIÓN PSIQUIÁTRICA EN CHILE: RELATOS DE LAS HOSPITALIZACIONES

La coerción psiquiátrica en las hospitalizaciones en Chile no se aleja mucho a la descripta en otros países. Cuestiones como restricciones a la libertad, control físico y psicofarmacológico son tónicas de estas experiencias, evaluadas negativamente por los usuarios en procesos de tratamiento psiquiátrico. En ese sentido, la hospitalización psiquiátrica está asociada a procesos de coerción señalados por varios usuarios que fueron parte de esta investigación. En sus narraciones emergen relatos coercitivos que se pueden interpretar como prácticas violentas relacionadas con sus conductas y que son percibidas con dolor y sufrimiento.

El entrevistado No.1, quien tiene un diagnóstico de esquizofrenia paranoide, indica frente al hecho de estar hospitalizado psiquiátricamente:

“*No deja de ser cruel estar hospitalizado ya sea en el Horwitz o en el Barros Luco, porque estar encerrado es igual que estar en una cárcel o un campo de concentración, la diferencia es que acá somos todos locos, supuestamente*”. (E1)

La referencia realizada por el usuario de la psiquiatría chilena homologa la experiencia de estar en un dispositivo psiquiátrico de encierro -como el hospital psiquiátrico Horwitz o la UHCIP del Hospital Barros Luco- con una cárcel o campo de concentración. Nos da a entender el tipo de vivencias de una persona en condiciones de encierro. En ese sentido, el encierro psiquiátrico se ha vuelto un lugar de coerción, es decir, un lugar donde la persona usuaria pierde toda libertad por decisión de especialistas en salud mental. En otras palabras, la hospitalización psiquiátrica como lugar del encierro es donde el paciente pierde la voz y, por tanto, la propia humanidad, tal como lo refiere el entrevistado al describirla como un campo de concentración, revalidando la tesis foucaultiana del vigilar y castigar, características de los dispositivos psiquiátricos del siglo XVIII y XIX^47^.

En esa misma línea, la entrevistada No. 5 (diagnosticada con trastorno afectivo bipolar) refiere sobre el disciplinamiento de la conducta al interior de estos lugares de encierro, donde la voluntad es redirigida a un modelo coercitivo:

“*Es tan horrible, uno lo pasa mal dentro. Te obligan a hacer cosas que tú no quieres, es como vivir una lógica militar, donde tu opinión no vale nada.* […] *La hospitalización es una cárcel donde uno no decide*”. (E5)

El encierro psiquiátrico representa un lugar de convivencia con la violencia simbólica, donde el paciente no puede hablar o tener una capacidad real de decisión frente al trabajador de la UHCIP u hospital psiquiátrico. La capacidad de decidir queda objetada por su condición. Como consecuencia, emergen emociones relacionadas con el sufrimiento, que determinan la forma de vivir esa experiencia de estar encerrado y privado de la libertad. A propósito de ello el entrevistado No. 6 (usuario diagnosticado con esquizofrenia) refiere:

“…*el tiempo de estar encerrado es un momento muy triste, es estar solo. Las personas que están dentro están igual que uno, tristes, con miedos incluso, pero que uno mismo debe tratar. Son los que están al cuidado de uno los que no son buenos, porque deben pensar que somos inferiores. Yo a ellos les temía, tenía miedo de que me hicieran algo porque veía cómo lo hacían con otros*”. (E6)

Las emociones que giran en torno al encierro estarían conectadas directamente con el miedo y lo traumático que significa estar en un lugar alejado de la libertad. No obstante, también se convierte en un lugar donde inmediatamente la persona se siente inferior al resto, o al menos en relación con los trabajadores de los dispositivos psiquiátricos, reconfirmando la tesis goffmaniana sobre los asilos^8^. La hospitalización psiquiátrica, según Mills^10^, en ese sentido, representa el lugar del miedo, el silencio, la inferioridad y el sufrimiento de quien cruza por esta experiencia traumática, en definitiva, es un lugar que está inmerso en la coerción.

Por otro lado, la experiencia del encierro psiquiátrico pone en tensión la cuestión de la decisión y la libertad, ya que al definirse como un dispositivo terapéutico que busca la compensación psicopatológica, la decisión de seguir hospitalizado no pasa por la persona en sí misma, es decir el usuario, sino más bien, por un equipo clínico-psiquiátrico que define la extensión de la estadía -o la detención- de la persona afecta. Barrera^39^ nos dice que el sujeto pierde toda capacidad de decisión al interior de un encierro psiquiátrico (o al menos con lo que tiene que ver con su libertad ciudadana). A propósito de lo anterior, el entrevistado No. 9 nos dice:

“*Mi experiencia de haber estado hospitalizado fue en el hospital clínico de la Universidad de Chile. Ahí fui un conejillo de indias. Me llevaron dopado a hospitalizarme, porque mi mamá me dio unos remedios a escondidas por orden del psiquiatra, me dormí, y no me di cuenta de nada. Desperté amarrado en una sala del hospital, decían que era por mi esquizofrenia, ya que era peligroso tener ese diagnóstico. Me hospitalizaron en contra de mi voluntad el año 2001. Después, despierto, veo que me están colocando cables en mi cabeza y me asusto. La cosa es que me estaban colocando cables para hacerme un electroschok. Fue terrible eso, porque yo reconocí la máquina y ni siquiera yo había aceptado eso, y tampoco se me había informado que me lo iban a realizar. La cosa es que yo dije: ‘a mí no me tienen que venir a hacer eso en la cabeza, yo no estoy loco cómo ellos dicen’. Después empiezo a sacarme los cables de mi cabeza y las enfermeras me gritan: ‘no se los saque’, pero ya me los había sacado casi todos cuando entonces me toman unos médicos o paramédicos y me inyectan y no supe más de mí nuevamente. Fue algo horroroso lo que viví, porque nadie nunca me dijo nada sobre hacerme un electroschok, y nunca nadie me dio una explicación sobre eso, de hecho, recuerdo poco lo que pasó ahí, y ya no quiero preguntar, fue muy traumático para mí estar hospitalizado en el psiquiátrico*”. (E9)

Este relato revela qué es vivir una experiencia relacionada con el encierro psiquiátrico desde la perspectiva de un usuario. Las prácticas coercitivas existentes se adhieren a las vivencias, y transforman al ser humano en lo más íntimo. Sin embargo y, por otro lado, también muestra la cara oculta de la psiquiatría: la violencia que puede significar una internación en contra de la voluntad de una persona^39^. 

El relato anterior recuerda las prácticas coercitivas más deshumanizadoras, y tal como planteaba el estudio de Inchauspe^40^, esa frase: “es por tu bien”, favorece una práctica coercitiva en una unidad que se define a sí misma como terapéutica. Ya sean médicos-psiquiatras, profesionales o técnicos, todos se ven envueltos en un engranaje coercitivo que funciona con normalidad y bajo la pérdida legal de libertad del individuo. El “no pedir permiso”, “no dar explicación”, el amarrar en condiciones de agitación -que en enfermería se denomina contenciones mecánicas^48^-, se convierten en modos de intervención validados en este campo. 

La coerción de las instituciones psiquiátricas se actualiza, contradictoriamente, en el propio proceso terapéutico ya que, de acuerdo a la norma técnica de la UHCIP^11^, la hospitalización conllevaría a la compensación psicoterapéutica y, por lo tanto, a un incremento en la calidad de vida del usuario. Sin embargo, las experiencias de usuarios lo muestran como una práctica en ocasiones violenta. En este sentido, el entrevistado No. 22 (diagnosticado con esquizofrenia) refiere:

“*Estar hospitalizado no fue una mala experiencia, ya que mis compañeros eran buenas personas, porque era gente que le pasaba lo mismo que a mí. Sin embargo, con los funcionarios era otra cosa, fue una mala experiencia. Ahora pienso que no me hospitalizaría más porque había humillación, y compañeros de tratamientos me cuentan que en las hospitalizaciones los funcionarios siguen maltratándonos*”. (E22)

La coerción en ocasiones podría expresarse también como violencia^24^ y esta estaría definida por la asimetría de las relaciones de cuidado^49^. Esta relación de poder se da entre una persona que necesita cuidados psiquiátricos y alguien que tiene esa capacidad técnica de entregar esos cuidados. Esto puede verse reflejado en una experiencia ocurrida en el hospital psiquiátrico Horwitz, donde el entrevistado No. 23 cuenta una historia marcada por la violencia:

“*Estaba hospitalizado el 2011 en avenida la Paz, y un compañero que llevaba dos meses se quería ir, e intentó salir de ahí, y los funcionarios le pegaron. A mí me dio miedo esa situación porque creo que eso no está bien. Él solo insistía que quería irse de ahí. Había muchos pacientes mirando esa situación, pero nadie podía interferir, yo creo que porque nos pasaría lo mismo* […] *esos funcionarios eran peligrosos, andaban como matones por los pasillos del hospital, yo les tenía miedo, te vigilaban siempre y te miraban a los ojos como si fueras a hacer algo malo. Incluso no dejaban que algunos de mis compañeros que eran cristianos, oraran o rezaran. En otra ocasión, recuerdo, ponían la camisa de fuerza a los que estaban más habladores y los amarraban en la cama, conmigo no lo hicieron, porque yo me portaba bien, pero lo hicieron con varios otros. Yo me preguntaba: ¿era necesario usar tanta brutalidad para eso?, pero nadie me iba a responder esa pregunta*”. (E23)

La internación psiquiátrica, indistintamente en qué tipo de unidad se practique, es una herramienta que busca la compensación psicopatológica de quienes están cursando alguna crisis. Sin embargo, en algunas ocasiones termina siendo un mecanismo coercitivo profundamente violento contra el cuerpo y las emociones de los pacientes. El miedo se axiomatiza en el individuo en este proceso terapéutico, transformándose en un lugar traumático, algo que el entrevistado No. 22 relata de acuerdo a su experiencia:

“*Por eso considero que no me hospitalizaría nuevamente, o sea, al menos no quiero vivir esa violencia. Yo soy un tipo muy tranquilo, más allá que tenga esquizofrenia, nunca le he hecho daño a nadie. Ahora claro, si estoy hospitalizado, y en algún momento me quisiera ir, deberían dejarnos ir. Pero sé que eso no ocurrirá hospitalizado, y no entiendo muy bien el por qué*”. (E22)

De acuerdo a lo anterior, la coerción psiquiátrica en la internación se erige como una forma de conservación del control y el disciplinamiento de las conductas de los pacientes psiquiátricos hospitalizados, y así el abuso con el “otro psiquiátrico” se convierte en una relación de poder en los espacios de cuidado. De acuerdo a los estudios de Völlm y Nedopi^50^, la privación de la libertad en cualquier institución de encierro es constitutiva de abusos, ya que se perpetúan malos tratos y violaciones de los derechos humanos de las personas. Para Girela^51^ el caso de las internaciones involuntarias es la más representativas, ya que en ella siguie operando la lógica del maltrato en espacios de cuidado, donde la contención mecánica se convierte en una de las prácticas coercitivas más importantes. En relación con lo anterior, Sailas y Fentom^52^ nos dicen que las contenciones mecánicas son la ausencia de lo terapéutico, algo que justamente entra en contradicción con el propósito terapéutico de las UHCIP o unidades de hospitalización. Para Méndez^53^ existe un uso abusivo en algunas unidades de hospitalización psiquiátrica de estas prácticas, comprendiéndolas incluso como prácticas de tortura y otros tratos crueles y abusivos. En relación con lo anterior, el entrevistado No. 24 (con diagnóstico de esquizofrenia) refiere:

“*Esto no se va a explicar de otra manera, el abuso es abuso y es una reacción que comete el más fuerte contra el más débil, y los que tenemos un problema psiquiátrico somos los más débiles. Y en un lugar como un hospital psiquiátrico, donde se debe ayudar a las personas, se abusa de ellas y se les maltrata, a mí me ocurrió, y además lo vi* […] *desde abusos verbales hasta golpes. Debe ser porque somos más vulnerables, porque somos esquizofrénicos, entonces todo el mundo cree que somos lo más bajo de la sociedad. Todos rechazan a quien tenga este tipo de enfermedad*”. (E24)

En consecuencia, se puede decir que el encierro psiquiátrico en muchas ocasiones es concebido como un elemento coercitivo en la experiencia de las personas con un problema psiquiátrico. Si bien esta no es percibida del mismo modo desde las políticas públicas, y de la propia psiquiatría que ha buscado más bien la regulación normativa, no ha existido una profundización en la relación terapéutica de esta intervención, y la búsqueda por el bienestar físico y psicoemocional de los individuos que han cursado la hospitalización psiquiátrica.

La razón experiencial del “loco” muestra cómo la internación psiquiátrica en muchas ocasiones se convierte en una práctica coercitiva y que no está exenta de abusos, deshumanización, tratos crueles e incluso golpes físicos y maltrato simbólico:

“*Hubo muy malas experiencias, me tuve que defender muchas veces de los abusos que cometían los funcionarios dentro del psiquiátrico. Mi experiencia en el COSAM* [Centro Comunitario de Salud mental] *es mucho mejor, pero hospitalizarse en sí es malo, quizás hace bien, no lo sé, pero es violento*”. (E5)

### El sufrimiento y la hospitalización psiquiátrica

Erwing Goffman^8^ es uno de los primeros que nos advierte acerca de la relación compleja de las hospitalizaciones psiquiátricas y los usuarios en dichas unidades, en su estudio sobre los internados en 1954. Este autor muestra cómo las instituciones totales -tales como los hospitales psiquiátricos- se convierten en lugares de sometimiento del paciente por parte del personal hospitalario, generando sumisión, obediencia y subordinación frente al personal hospitalario. Goffman nos dice que los hospitales psiquiátricos, como instituciones totales, terminan siendo “invernaderos donde se transforma a las personas”^8^ y esa transformación está amparada bajo un mecanismo coercitivo que favorece el sufrimiento de estas personas y que siempre está asociado a la mortificación del yo^8^

Las personas que han atravesado esta experiencia confirman de algún modo lo que Goffman nos ha venido diciendo desde hace casi más de sesenta años. Más allá de ser un hospital psiquiátrico o una unidad de hospitalización psiquiátrica en un hospital general, lo que se vive alrededor y al interior de ellos, reflejan experiencias complejas de sufrimiento.

Para Emmanuel Levinas, el sufrimiento se constituye en denegación y rechazo de una cualidad sensible que opera sobre el modo en que el sujeto experimenta la sensación, en otras palabras “…la forma en la que, en la conciencia, lo insoportable no puede soportarse”^54^. Para el autor, la conciencia no es algo activo, ya que en sí mismo el sufrimiento es pasividad pero, al mismo tiempo, es un “padecer la adversidad, incluso padecer el padecer”^54^. Esto último se debe a que la conciencia es consciente de su dolor, ya que está definido por su adversidad misma, es decir, su mal. El sufrimiento “por tanto” es el mal por el cual el sujeto camina pasivamente y en el que la sensibilidad es pura vulnerabilidad^54^. En este sentido, el sufrimiento se comprendería mediante el padecer que se traduce a través de un mal al que el sujeto se ve enfrentado, Levinas dirá: “La humanidad del hombre que sufre se halla abrumada por el mal que lo desgarra”^54^ y que puede estar asociado a una cárcel, a un encierro, es decir, una no libertad.

En esta investigación, algunas personas usuarias indicaron que la hospitalización tiene significaciones positivas, es decir, creen que la hospitalización beneficia cuando están cursando una crisis y que, por tanto, a largo plazo, tienen efectos buenos en sus vidas. Sin embargo, muchos aclaran que una cosa es el beneficio y otro es el hecho de estar hospitalizado o encerrado, es decir privado de la libertad, y el miedo que ello produce. El entrevistado No. 8, un joven diagnosticado con esquizofrenia, nos dice:

“…*es que la imagen de un hospital psiquiátrico que tengo es el hospital psiquiátrico Horwitz, y es lo más tenebroso en lo que he estado. Recuerdo que cuando estuve una vez ahí, porque tenía alucinaciones místico-religiosas, me amarraron y me inyectaron, después pasé días ahí y nadie me explicaba nada* […] ¿Es así el Peral?”. (E8)

La cuestión del sufrimiento -tal como Levinas lo plantea- es algo que estaría en el mundo interior de las personas que cursan la experiencia de ser hospitalizadas psiquiátricamente. La condición diagnóstica y la subjetividad humana se conjugan en la relación usuario-trabajador, teniendo efectos performativos y transformando emocionalmente la vida del sujeto diagnosticado y en condición de encierro. Ello lo manifiesta el entrevistado No. 9, persona diagnosticada con depresión severa:

“*Yo me hospitalice voluntariamente, y recuerdo una vez que le pregunté algo a una enfermera o paramédico -no recuerdo bien eso-, me retó porque eso no debía preguntárselo a ella. La verdad no entendía muy bien lo que pasaba ahí adentro, entonces me comencé a sentir triste, así como abandonado*”. (E9)

La narrativa del sufrimiento puede distinguirse en la soledad, la tristeza y el dolor, que en un principio se asocia a lo psicopatológico, y que emerge como una consecuencia radical del abandono en un espacio de cuidado. El hecho de estar apartado de la sociedad, y vinculado a un establecimiento psiquiátrico público, en el que la voz del sujeto queda silenciada en actos cotidianos, se convierte en la principal arma de destrucción del yo, en términos goffmanianos. El entrevistado No. 2 (usuario con diagnóstico de esquizofrenia) nos dice al respecto:

“*En el 2010, al principio, cuando me iban a ver los doctores en el Horwitz, pasaban como en una junta médica día por medio para revisarme. Yo sentía que ellos pensaban que era un espécimen que tiene esto y esto otro. Es ahí que ellos mencionan el trastorno que tenía y me causó impacto. ¿Qué tendré?, me preguntaba, o ¿qué cosa tengo? Me asusté un poco, pero en mi mente pensaba que me tenía que mejorar. Nadie me explicó nada, y yo me asusté*”. (E2)

Al interior de las hospitalizaciones psiquiátricas, la vida cotidiana funciona con una constante y persistente despersonalización de los sujetos. Nadie explica nada y los internos se convierten en objetos que son manipulados y manejados de modos insospechados. No obstante, estos mismos objetos de la psiquiatría cuentan sus experiencias de dolor y miedo en estos lugares, alejados de cualquier tratado internacional de derechos humanos o leyes que los resguarden. La entrevistada No. 8, refiere lo que ocurre dentro de estos espacios:

“*Estar tantas veces internada en el psiquiátrico, de alguna manera hizo que yo aprendiera los códigos de cómo funcionaba el hospital psiquiátrico, ahí aprendí desde que la levantan a uno hasta qué es lo que se tiene que hacer y qué no tiene que hacer* […] *nadie te explica nada, es decir, todos los que trabajan ahí, solo te miran, te hacen preguntas y se van, y no entiendes nada. Y si uno pregunta, se molestan*”. (E8)

La forma en que se relaciona el personal con los internos representa la indiferencia frente al sufrimiento psíquico. Anderson^55^ nos dice que el sufrimiento estaría muy asociado a un dolor del alma y este está muy relacionado con las experiencias existenciales de las personas. De esa manera, en los dos relatos anteriores se puede visualizar cómo el sufrimiento está inscrito en una relación displicente con el “loco”, como si fuera un objeto sin valor y sin voz. El sufrimiento se manifiesta, por un lado, en la relación de indiferencia entre el personal y la persona^8^ que está internada en el hospital por un problema psíquico y, por otro lado, emerge como una forma de control de conductas, en la que el hecho de ser paciente psiquiátrico elimina cualquier capacidad de relación democrática, es decir, es como que perdiera todo derecho por el solo hecho de estar en una condición de encierro. Así, la hospitalización emerge como un mecanismo autoritario y coercitivo que violenta y absorbe al interno. Frente a lo anterior, la entrevistada No. 7 (usuaria con diagnóstico de trastorno afectivo bipolar) nos dice:

“*O sea, lo que a mí me sucede* [hospitalizada] *cuando me descompenso, es que me quiero ir de ahí, pero anularon mi voluntad. Ahora que lo pienso bien, quería irme y no podía, me lo impedían, y lo que ocurre después es la reacción normal -creo- de querer irme a mi casa, estar libre y, después de eso, me amarran, me inyectan y despierto un día después, y eso es traumático*”. (E7)

Cuando Levinas indica que el ser humano sufre porque se encuentra abrumado por el mal que lo desgarra, se refiere justamente a la asociación entre el encierro y el campo de concentración (como fue en el caso del autor), es decir, a la pérdida de la libertad. Al parecer, el sufrimiento psíquico de quienes han sido diagnosticados psíquicamente se asocia a la pérdida de la libertad en la internación psiquiátrica, ya que ahí el cuerpo del sujeto internado queda disponible en una relación de poder. En esa línea, el entrevistado No. 10 nos dice: “*se ven cosas malas en la hospitalización, uno sufre ahí*” (E10) y, en consecuencia, el lugar del cuidado se vuelve un sector del lamento y del dolor. Es en ese sentido que muchos declaran no querer repetir tal experiencia, porque justamente es apelar a la vuelta al dolor continuo y la exposición al maltrato: “…no me hospitalizaría [otra vez] […] me han contado varios compañeros que los maltratan en el psiquiátrico…” (E10). Y las propias experiencias de los protagonistas de estas historias reafirman que la violencia y la tortura se constituyen como cuestiones legitimas al interior de estos lugares:

“…le pegaron a un paciente, y los gallos no me gustaron […] eran peligrosos esos enfermeros, como que siempre andaban como matones adentro de las salas del hospital”. (E10)

## DISCUSIÓN

Las formas de coerción psiquiátrica emergen en los relatos de las personas que han cursado la internación psiquiátrica^23^, asociándose al miedo y el castigo, en definitiva, al sufrimiento, significando experiencias negativas en estos espacios. Los relatos que se recogieron en esta investigación lo muestran con detalle, al menos en términos narrativos, como forma analítica. 

Para Philip Fennel^56^, lo anterior se convierte en una práctica de violencia y coerción, y estas son características de los lugares de reclusión y encierro. Se podría decir que, la hospitalización se constituye como un poder clínico (psiquiátrico) que funciona coercitivamente en las personas, doblegando la voluntad de los sujetos afectos a esta condición de encierro:

“…la detención, el tratamiento forzado sin consentimiento, la reclusión y la restricción son los principales mecanismos de poder clínico. Una persona puede consentir el tratamiento o permanecer en el hospital si sabe que se verá obligada en caso de rechazo. El cumplimiento a la sombra de la compulsión es una característica importante del sistema psiquiátrico”.^56^

De algún modo, la coerción psiquiátrica queda vinculada al sufrimiento psíquico, a través de la violencia que esta utiliza de manera directa e indirecta. Pero, por otro lado, la coerción también queda anclada al miedo a la hospitalización psiquiátrica, a pesar de que esta pudiera ser necesaria, algo que narran en sus historias los usuarios de la salud mental. 

Como Chile no tiene otras alternativas para tratar las crisis de las personas que cursan diagnósticos psiquiátricos, la internación en dispositivos de encierro se establece como la única vía de tratamiento, o la principal al menos en periodos de crisis. Sin embargo, el miedo que genera una hospitalización psiquiátrica, por su coercitividad al interior de estos espacios clínicos, favorece los estigmas hacia este tipo de dispositivos. Los estudios de Gooding, McSherry, Roper y Grey^5^ reafirman que la coerción psiquiátrica -especialmente en la internación- generan miedo y aumento de sujeción. Siguiendo esa línea, Castro nos dice que el miedo a la coerción puede mantener a las personas alejadas del tratamiento de salud mental, lo que puede aumentar el riesgo de coerción^2^. A pesar de que los trabajadores de la psiquiatría involucrados en estos temas desaprueban teóricamente la coerción, en la práctica, terminan fomentando este tipo de intervenciones, tal como lo muestran los relatos presentados. Esto último confirma lo que Gooding^37^ indica a propósito de los efectos de la coerción, ya que la privación de la libertad y/o la detención psiquiátrica favorece el abuso hacia el “loco”. 

Son las propias personas usuarias de la psiquiatría quienes se dan cuenta que existen injusticias al interior del internamiento psiquiátrico, y ellas mismas son quienes experimentan este sufrimiento, las que narran en primera persona el trato indigno que se da. La experiencia dolorosa que se obtiene en la internación psiquiátrica puede tomar ribetes mucho más complejos; tanto así, que, al suspender los derechos, ya sea de manera legal o ilegal, hacen ocupar una posición inferior al sujeto “loco”. Como diría Bourdieu: “esa miseria de posición”^57^, que experimenta el sujeto diagnosticado, que hunde en lo más profundo del abismo cualquier humanidad. 

De esa manera, la importancia del abordaje de las prácticas de intervención en las hospitalizaciones psiquiátricas se vuelve importante a la luz de los antecedentes de coerción existentes. Los esfuerzos que se realizan en distintos lugares del mundo deben servir para fomentar el enfoque de derechos y prácticas más humanizadoras en Chile, con quienes cruzan episodios críticos de su salud mental en el nivel público. Esto se vuelve importante, ya que justamente para bajar los niveles de coerción psiquiátrica existentes en las unidades de hospitalizaciones se necesitan investigaciones aplicadas que den paso a repensar prácticas basadas en derechos. De ese modo, avanzar hacia procesos de construcción de una nueva cultura de derechos en las prácticas de intervención de las instituciones psiquiátricas y de salud mental en nuestro país se vuelve fundamental y prioritario.

## CONCLUSIONES

Las consecuencias que tiene la coerción en el contexto de la hospitalización psiquiátrica en Chile han sido muy similares a las que los estudios citados en este trabajo han mostrado. La falta del enfoque de derecho, como una realidad experiencial, ha traído consigo una perpetuación de prácticas coercitivas en nuestro país, evidenciable en los relatos de usuarios de la salud mental chilena. Tal como plantea Rzhevskaya^43^, estas prácticas se instalan con una violencia imperecedera que se han encarnado a través de la internación psiquiátrica de nuestro país. La pérdida de libertad en estos espacios “terapéuticos”, además del menoscabo de la voz del usuario y la deshumanización, han fomentado el sufrimiento, el miedo y el trauma, además de los sentimientos de inferioridad de los usuarios en estos lugares. 

Pensar la hospitalización psiquiátrica desde un enfoque de derechos, como horizonte posible de intervención, podría fundamentar formas más democráticas para alcanzar el bienestar social, físico y psicoemocional de los usuarios de la salud mental chilena. Sin embargo, una de las dimensiones más importantes que muestra este trabajo es que las consecuencias de la coerción psiquiátrica, en el caso de las hospitalizaciones, muestran una escasa mejoría en los usuarios, un impacto negativo y la ausencia de libertad ciudadana. Las prácticas forzosas de procedimientos como las contenciones mecánicas o psicofarmacológicas han ido en desmedro de los usuarios de la salud mental y psiquiatría, provocando un menoscabo en las emociones y sentimientos de estos. La lógica del “es por tu bien” se reafirma más como una práctica de poder, en la que el más débil es el paciente psiquiátrico, favoreciendo el sufrimiento psíquico, ya no como una perspectiva psicopatológica, sino de una práctica gubernamental de ejercicio del poder, que aplasta las emociones de la persona que cursa un problema de salud mental en la realidad chilena.

Es importante generar una política de reducción de la coerción psiquiátrica, en la que se incorporen a las personas usuarias con experiencias en primera persona en este campo, ya que la falta de inclusión de estas personas en la discusión va en menoscabo de la mejoría en estos espacios “terapéuticos”. Eliminar la lógica “es por tu bien”, generaría un impacto positivo que reduciría las prácticas coercitivas, con un énfasis fundamental en la perspectiva de derechos humanos y una salud mental más democrática.

## References

[1] Bevía B, Girón M (2017). Poder, estigma y coerción: Escenarios para una práctica no autoritaria en salud mental. Revista de la Asociación Española de Neuropsiquiatría.

[2] Castro MA (2021). Los efectos performativos de la psiquiatría en la vida de las personas diagnosticadas psiquiátricamente: el sufrimiento de la locura.

[3] Castro MA (2020). El sufrimiento psíquico de las personas con un diagnóstico psiquiátrico, el dolor de la locura. Revista Perspectivas.

[4] Ardiles S (2019). En nombre Propio: Relatos de vida de mujeres que tuvieron internaciones psiquiátricas prolongadas y ahora viven en la comunidad.

[5] Gooding P, Mc Sherry B, Roper C, Grey F (2018). Alternatives to coercion in mental health settings: a literature review.

[6] Bevía B, Del Trigo AB (2017). Coerción y salud mental.

[7] Gobierno de Chile (2021). Ley 21.331 del Reconocimiento y Protección de los Derechos de las Personas en la Atención en Salud Mental.

[8] Goffman E (1998). Internados: Ensayos sobre la situación social de los enfermos mentales.

[9] Fennel P, Mc Sherry B, Weller P (2010). Rethinking rights-based mental health laws.

[10] Mills C (2014). Decolonizing global mental health, the psychiatrization of the majority world.

[11] Subsecretaría de Redes Asistenciales (2016). Modelo de gestión, unidad de hospitalización de cuidados intensivos en psiquiatría para población adulta e infanto-adolescentes.

[12] Gobierno de Chile (2017). Política y Plan Nacional de Salud Mental 2017-2025.

[13] Naciones Unidas (2016). Convención sobre los derechos de las personas con discapacidad: Observaciones finales sobre el informe inicial de Chile.

[14] Instituto Nacional de Derechos Humanos (2018). Informe Anual sobre Derechos Humanos en Chile.

[15] Corte Suprema de Chile (2018). Oficio Nº 164-2018, Informe Proyecto de Ley Nº 40-2018.

[16] Cea JC (2018). Por el derecho a la locura: La reinvención de la Salud Mental en América Latina.

[17] Instituto Nacional de Derechos Humanos (2017). Informe Anual Situación de los Derechos Humanos en Chile.

[18] Gobierno de Chile (2018). Modelo de gestión de la red temática de salud mental en la red general de salud.

[19] Desviat M, Moreno A, Desviat M, Moreno A (2012). Acciones de la salud mental en la comunidad.

[20] Gøtszche P (2016). Psicofármacos que matan y denegación organizada.

[21] Huertas R (2017). Políticas de salud mental y cambio social en América Latina.

[22] Fernández Liria A (2018). Locura de la psiquiatría, apuntes para una crítica de la psiquiatría y la “salud mental”.

[23] Ortiz-Lobo A, Huertas R (2018). Críticas y alternativas en psiquiatría.

[24] Steintert T, Leppig P, Berhardsgrütter R, Conca A, Hatlin T, Janssen W (2010). Incidence of seclusion and restraint in psychiatric hospitals: a literature review and survey of international trends. Social Psychiatry and Psychiatric Epidemiology.

[25] Siponen U, Välimäki M, Kaltiala-Heino R (2011). A comparison of two hospital districts with low and high figures in the compulsory care of minors: An ecological study’. Social psychiatry and psychiatric epidemiology.

[26] Ulla S, Maritta V, Riittakerttu KH (2012). The use of coercive measures in adolescent psychiatric inpatient treatment: a nation-wide register study. Social Psychiatry and Psychiatric Epidemiology.

[27] Andersen K, Nielsen B (2016). Coercion in psychiatry: the importance of extramural factors. Nordic Journal of Psychiatric.

[28] Flammer E, Steintert T (2016). Association between restriction of involuntary medication and frequency of coercive measures and violent incidents. Psychiatric Service.

[29] Barnicot K, Insua-Summerhayes B, Plummer E, Hart A, Barker C, Priebe S (2017). Staff and patient experiences of decision-making about continuous observation in psychiatric hospitals. Social Psychiatry and Psychiatric Epidemiology.

[30] Henderson C, Flood C, Leese M, Thornicroft G, Sutherby K, Szmukler G (2004). Effect of joint crisis plans on use of compulsory treatment in psychiatry: single blind randomised controlled trial. The British Medical Journal.

[31] Maylea C (2017). Coerciveness in coercion: A case study of social work powers under the victorian mental health. Australian Social Work.

[32] Fletcher J, Spittal M, Brophy L, Tibble H, Kinner S, Elsom S, Hamilton B (2017). Oucomes of the victorian safewards trial in 13 wards: Impact on seclusion rates and fidelity measurement. International Journal of Mental Health Nursing.

[33] Sampogna G, Luciano M, Del Vecchio V, Pocai B, Palummo C, Fico G (2019). Perceived coercion among patients admitted in psychiatric wards: Italian results of the EUNOMIA Study. Front Psychiatry.

[34] Goulet MH, Larue C, Lemieux AJ (2018). A pilot study of “post-seclusion and/or restraint review” intervention with patients and staff in a mental health setting. Perspectives in Psychiatric Care.

[35] Mann-Poll P, Smit A, Noorthoorn EO, Janssen WA, Koekkoek B, Hutschemaekers Giel JM (2018). Long-term impact of a tailored seclusion reduction program: Evidence for change?. Psychiatric Quarterly.

[36] Thomsen CT, Benros ME, Hastrup LH, Andersen PK, Giacco D, Nordentoft M (2018). Patient controlled hospital admission for patients with severe mental disorders: A nation wide prospective multicentre study. Acta Psychiatrica Scandinavica.

[37] Gooding P, Mc Sherry B, Roper C, Grey F (2018). Alternatives to coercion in mental healthsettings: a literature review.

[38] Bustamante JA, Cavieres A (2018). Internación psiquiátrica involuntaria: antecedentes, reflexiones y desafíos. Revista Médica de Chile.

[39] Barrera A (2021). Toda ley de salud mental debe legislar sobre la hospitalización involuntaria, o no será efectiva. Cuadernos Médicos Sociales.

[40] Inchauspe JA (2019). Actualidad en coerción y asistencia en salud mental: de la pugna declarativa al impulso a alternativas. Revista de la Asociación Española de Neuropsiquiatría.

[41] Bottlender R, Juckel G (2019). Coercion and aggression in psychiatry: the individual psychological dimension of aggressive and coercive acts by therapists. Fortschritte der Neurologie Psychiatrie.

[42] Cea JC, Castillo T (2020). Electroshock o Terapia Electroconvulsiva (TEC) en Chile: Diagnóstico crítico, activismo social y enfoque de derechos. Quaderns de Psicología.

[43] Rzhevskaya N, Ruzhenkov V, Ruzhenkova V, Khamskaya I, Moskvitina U (2020). Psychiatric coercion and violence: ethical, legal and preventive aspects. Archivos Venezolanos de Farmacología y Terapéutica.

[44] Cocho C, Vera I, Bardón B (2021). Satisfacción percibida con los ingresos en unidades de hospitalización breve psiquiátricas: diseño y validación del cuestionario PSYQUEST. Revista de Psiquiatría y Salud Mental.

[45] Lasllet B (1999). Personal narratives as sociology. Contemporary Sociology.

[46] Chase S, Denzin N, Linconl Y (2015). Métodos de recolección y análisis de datos: Manual de Investigación Cualitativa. Volumen IV.

[47] Foucault M (2002). Vigilar y castrigar: nacimiento de la prisión.

[48] Tekkas K, Bilgin H (2010). Professional containment methods used in psychiatry wards: Justifications for their utilization, types, international practices, and perceptions. Turk Psykiyatri Dergisi.

[49] Leal M, Santos F (2012). El tratamiento ambulatorio involuntario: Historia de una obstinación. Revista de la Asociación Española de Neuropsiquiatría.

[50] Völlm B, Nedopi N, Völlm B, Nedopi N (2016). The use of coercive measures in forensic psychiatric care: Legal, ethical and practical challenges.

[51] Girela E, López A, Ortega L, De Juan J, Ruiz F, Bosch J, Barrios L (2014). Variables associated with the use of coercive measures on psychiatric patients in Spanish penitentiary centers. BioMed Research International.

[52] Sailas E, Fenton M (2000). Seclusion and restraint for people with serious mental illnesses. Cochrane Database of Systematic Reviews.

[53] Unidas Naciones (2013). Informe del Relator Especial sobre la tortura y otros tratos o penas crueles, inhumanos o degradantes, Juan E. Méndez.

[54] Levinas E (2001). Entre nosotros, ensayos para pensar en Otro.

[55] Anderson R (2014). Human suffering and quality of life, conceptualizing stories and statistics.

[56] Fennel P, Mc Sherry B, Weller P (2010). Rethinking rights-based mental health laws.

[57] Bourdieu P (1999). La miseria del mundo.

